# Sex bias in prospective follow-up observational studies with drugs carried out in a southern region of Europe

**DOI:** 10.3389/fphar.2024.1427293

**Published:** 2024-11-01

**Authors:** Àlex Santomà, Anna Maria Jambrina, Adela Perisé, Mercè Armelles, Lluisa Perisé, Clara Pareja, Neus Rams, Manel Rabanal

**Affiliations:** ^1^ Directorate-General for Healthcare Planning and Regulation, Ministry of Health, Government of Catalonia, Barcelona, Spain; ^2^ Physiology Section, Department of Biochemistry and Physiology, Faculty of Pharmacy and Food Science, University of Barcelona, Barcelona, Spain

**Keywords:** sex bias, prospective follow-up observational studies, observational studies with drugs, female underrepresentation, gender bias, patient selection

## Abstract

**Introduction:**

The impact of sex bias in medical research is a matter of significant relevance and importance especially in the modern age. Despite notable improvements in sex equity across various societal fields, disparities in sex representation persist within clinical and pharmacological research. The objective of this article is to investigate the sex bias within Prospective Follow-up Observational Studies with Drugs authorized by the Advisory Commission on Post-Authorization Studies with Medicines in Catalonia, a southern European region.

**Methods:**

A retrospective study that analyses data from final reports of Prospective Follow-up Observational Studies with Drugs authorized by the Advisory Commission on Post-Authorization Studies with Medicines in Catalonia from 2015 to 2021. Disease categories and specific diseases, obtained from the Global Data Exchange, were evaluated for sex bias, comparing female participation to female prevalence.

**Results:**

There were 1,06,399 participants, including 43,778 female participants (42.5%). A significant underrepresentation of females was observed across various disease categories. Notably, in 12 out of 19 categories (63.2%), a pronounced female underrepresentation (sex bias ≤ 0.05) was evident, particularly in the categories of HIV/AIDS and sexually transmitted infections (sex bias = −0.5659). Furthermore, 11 categories (57.9%) also demonstrated significant female underrepresentation, with the same notable categories, HIV/AIDS and sexually transmitted infections (sex bias = −0.4439). When examining specific diseases, significant female underrepresentation was observed in 13 out of 29 diseases (46.4%), especially in HIV (sex bias = −0.4781). The overall findings indicate that the degree of sex bias was notably less favorable for females in numerous disease categories and specific conditions.

**Conclusion:**

Our study has demonstrated a significant sex bias within observational studies, mirroring patterns observed in clinical trials. Importantly, our findings highlight a pervasive underrepresentation of women across various disease categories and specific conditions. Despite efforts to promote both sexes inclusivity, our results emphasize the persistent challenges in achieving balanced sex representation in study populations. Furthermore, the absence of categorization of diseases based on male and female prevalence poses a significant challenge in accessing pertinent data, particularly concerning the sex distribution of specific diseases.

## 1 Introduction

The impact of sex bias in medical research is a matter of relevance and importance in especially in the modern age. Despite notable improvements in gender equity across various societal areas, disparities in sex representation persist within clinical and pharmacological research, resulting in disparities in the treatment received by female patients (Clayton and Tannenbaum, 2016; Willingham, 2022; Barlek et al., 2022). As medicine evolves toward a more personalized and evidence-based approach, addressing sex bias becomes imperative to ensure robust scientific outcomes and optimal care for all individuals.

The field of medical research is huge and multifaceted, containing a wide range of study designs aimed at exploring the efficacy, safety and outcomes of various medical interventions. Among these, observational studies with medications stand as a critical pillar in the pursuit of comprehensive evidence. These studies, often conducted in real-world settings, offer valuable insights into the effects of drugs under conditions that more closely mirror everyday clinical practice (BOE, 2020).

The exclusion of women from clinical trials and observational studies was historically justified for safety and simplicity. This is due to the tragedy of the teratogenic effect of thalidomide in the 60’s, and this has resulted in substantial gaps in our understanding of how medical treatments affects different sexes (Clayton and Tannenbaum, 2016). However, ensuring the proper application of clinical study findings requires the inclusion of participants that accurately represent the intended treatment population (Feldman et al., 2019). Neglecting the inclusion of underrepresented sexes can undermine the external validity of study results, impeding the translation of research into clinical practice and potentially perpetuating sex-related disparities in healthcare outcomes.

To counteract this situation, the National Institutes of Health (NIH) in 1993 changed the model that excluded women from the phase III of clinical trials, recommending their inclusion (Sundari Ravindran et al., 2020). Following this recommendation, most of the regulatory agencies of America, Canada and Europe have been working to include sex in the regulation of the pharmaceutical research, based on the influence in the efficacy and safety of the medicines (Willingham, 2022; Sundari Ravindran et al., 2020). Since 2016, the NIH has recommended the inclusion of sex as a variable in all research protocols it funds (Woitowich and Woodruff, 2018). Despite this, by 2015 fewer than one-third of evaluated NIH-funded randomized controlled trials were including both sexes in their studies or providing an explanation for not doing so (Willingham, 2022).

Furthermore, the lack of objective data complicates the prescription of medicines for pregnant woman. An analysis conducted using the Clinical Trials database, which includes phase IV studies promoted by the pharmaceutical industry in the United Sates, showed that 95% of studies on drugs that have no teratogenic potential excluded pregnant women, and only 1% were specifically designed for pregnant patients. According to the authors, pregnant women should be included in phase IV studies with medications, as their systematic exclusion has a negative impact on their health (Institute for Health Metrics and Evaluation, 2023), a viewpoint shared by some gynecology and obstetrics specialists in the United States (Briggs et al., 2015). Incorporating pregnant women into clinical research would likely enhance the evidence base for making informed treatment decisions during pregnancy, ultimately leading to better health outcomes for both women and children. Therefore, the benefits of this inclusion could manifest both in the short term and in the long term, depending on the specific illness (Shields and Lyerly, 2013).

In light of these developments, the aim of this article is to investigate the sex bias within Prospective Follow-up Observational Studies with Drugs (EOm) authorized by the Advisory Commission on Post-Authorization Studies with Medicines in Catalonia (CAEPAM), a southern European region. Examining the roots of sex disparity and its repercussions for both research and clinical practice could help in implementing strategies to mitigate its impact.

## 2 Material and methods

### 2.1 Data sources

We conducted a retrospective descriptive study using data collected from final reports and published articles provided by sponsors of EOm authorized by the CAEPAM, within the period from 2015 to 2021. The selected EOm for this analysis excluded those focusing on sex-exclusive disease, such as prostate cancer or antiemetic used in pregnancy studies. We omitted those that did not involve any drug, as CAEPAM cannot evaluate them, since this commission only assesses observational studies involving medications. We excluded also those EOm that did not segregate the participants by sex. It is worth noting that some of the observational studies did not focus on any disease, and we did not include these in our study.

Global disease prevalence data for female participants were obtained from the Global Data Exchange (GHDx), a database synthesized from multiple data sources, including scientific literature and population representative surveys (Institute for Health Metrics and Evaluation, 2023; GBD 2016 Causes of Death Collaborators, 2017). Prevalence values for selected disease’s category and specific diseases defined by GHDx were obtained from an online catalog of health-related data (Institute for Health Metrics and Evaluation, 2023).

### 2.2 Variables

The sex bias analysis has been divided into disease’s categories (n = 19) and specific diseases (n = 28), in alignment with the categories present in the GHDx international database (Institute for Health Metrics and Evaluation, 2023). These are listed in Tables 1, 2, respectively.

**TABLE 1:** Disease’s categories included on this study.

Disease’s category
Cardiovascular diseases
Chronic respiratory diseases
Diabetes and kidney diseases
Digestive disease
Endocrine, metabolic, blood and immune disorders
HIV/AIDS and sexually transmitted infections
Maternal and neonatal disorders
Mental disorders
Musculoskeletal disorders
Neoplasm
Neurological disorders
Other infectious diseases
Respiratory infections and tuberculosis
Sense organ diseases
Skin and subcutaneous diseases
Substance use disorders
Total burden related to hepatitis B
Total burden related to hepatitis C
Urinary diseases and male infertility

**TABLE 2:** Specific diseases included on this study.

Specific disease
Acute myeloid leukemia
Adenocarcinoma gastric
Alcoholism
AMD (Age-related macular degeneration)
Asthma
Atopic dermatitis
Atrial fibrillation
Breast cancer
Chronic ischemic cardiovascular disease
Colorectal cancer
COPD (Chronic obstructive pulmonary disease)
Epilepsy + drowsiness
Hepatitis B
Hepatitis C
HIV (Human immunodeficiency virus)
Infections in neonates
Inflammatory bowel disease
Major depression
Migraine
Multiple myeloma
Multiple sclerosis
Open-angle glaucoma/ Ocular hypertension
Parkinson’s disease
Psoriasis
Renal cell carcinoma
Rheumatoid arthritis
Schizophrenia
Type 2 diabetes

Female prevalence fraction (F-Prev) for each disease’s category and specific disease was defined as the fraction of female participants in each aggregation, and was estimated by dividing the global morbidity count for female participants by global morbidity count for both male and female participants using GHDx (Institute for Health Metrics and Evaluation, 2023). The data obtained from this database dates from 2019.

Female participant fraction (F-particip) was defined as the fraction of female participants among all participants who were included in the EOm, and was estimated in two ways:

a) “Studies” as measurement units, by calculating the ratio of female participants to all participants for each EOm and determining the simple average of this ratio for all EOm without any weighting by EOm size.
b) “Participants” as measurement units, by dividing the total number of female participants in all EOm by the total number of male and female participants in all EOm combined.

The female participant fraction was estimated from the final reports provided by the sponsor.

The main focus was on sex bias in enrollment in EOm, defined as the difference between female participation (F-particip) and female prevalence (F-Prev). Sex bias values ranged from −1 to 1, with 0 indicating no bias. A negative sex bias means that female participants were less represented than male participants. Female underrepresentation was considered when the sex bias was equal to or less than −0.05. Conversely, a sex bias equal to or greater than 0.05 indicated female overrepresentation.

## 3 Results

**FIGURE 1:**
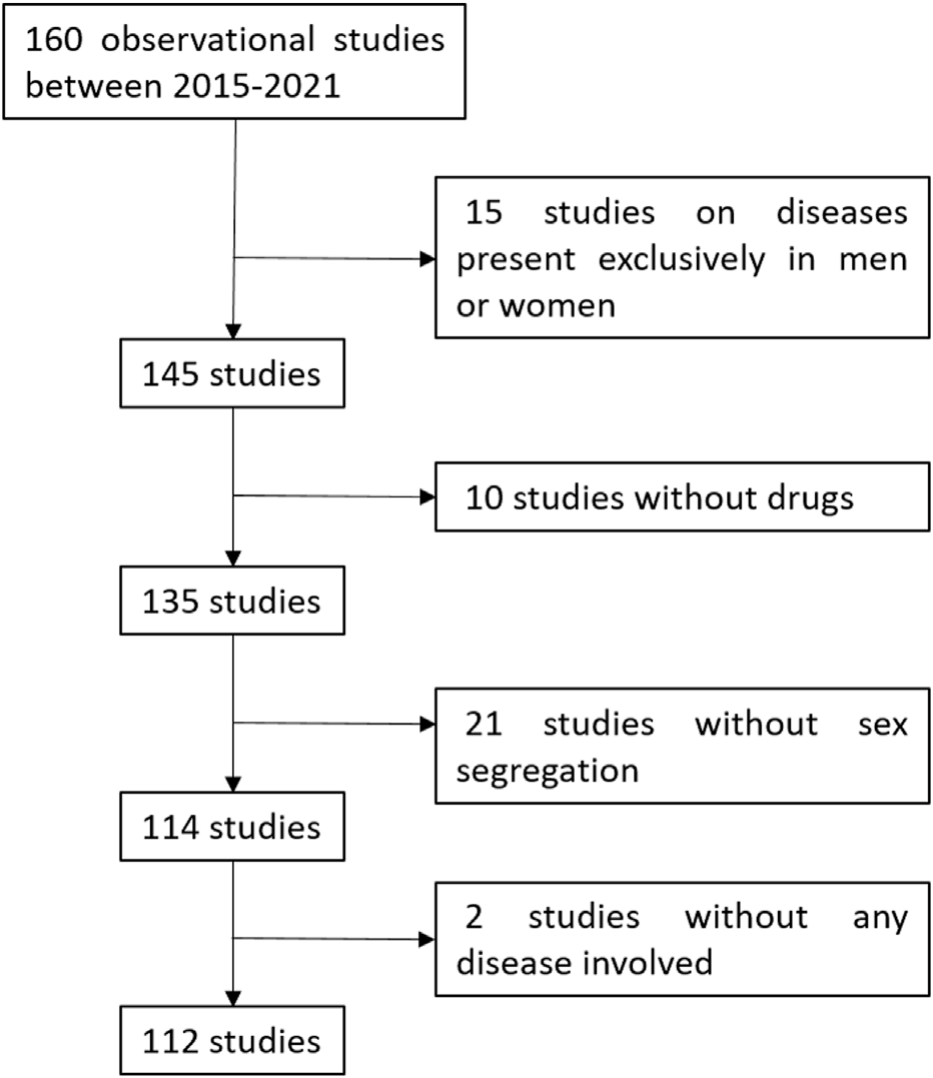
Algorithm of the studies included and excluded on this review.

There were 160 EOm authorized by CAEPAM from 2015 to 2021 that have finished and have the final report. Of these, 15 EOm (9.4%) were conducted on diseases present exclusively in men or women. Additionally, there were 10 EOm (6.3%) with no drug involved, 21 EOm (13.1%) did not included sex segregation. Finally, there were 2 EOm (1.3%) that did not involve any disease (Figure 1).

Therefore, of the 160 studies for which the final report was available, we included 112 (70.0%) in our study.

### 3.1 Descriptive data of EOm

Out of the 112 analysed EOm, 40.2% correspond to international studies, whereas 59.8% pertain to studies exclusively conducted within the Spanish territory (Table 3). The total number of patients included in the 112 analysed EOm is 106,399. Overall, males constitute 57.5% of the included patients, while females make up the remaining 42.5%, using “participants” as a measurement unit. Using “studies” as a measurement unit, the percentage of women included rises up to 45.3%.

**TABLE 3:** Differences of sex segregation between national and international studies.

Ambit	Total studies (N)	Total studies (%)	Women (N)	Men (N)	Participants (N)	Measurement unit	Females (%)
International	45	40.2	36,262	48,644	84,906	Studies	48.5
						Participants	42.7
National	67	59.8	7,516	13,977	21,493	Studies	43.1
						Participants	43.2
Total	112	100.0	43,778	62,621	1,06,399	Studies	45.3
						Participants	42.5

### 3.2 Category disease

**FIGURE 2:**
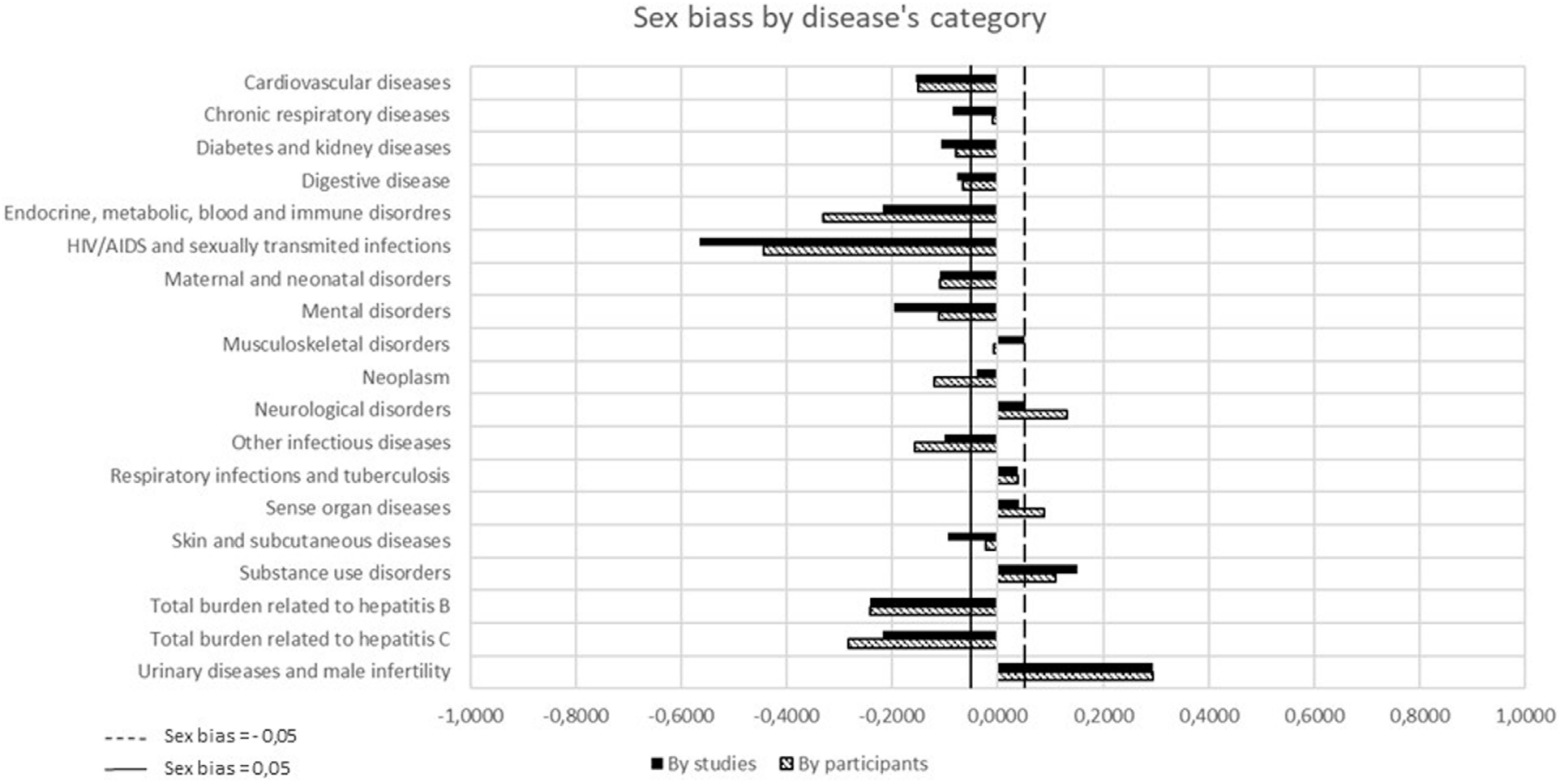
Sex bias by category disease.

Table 4 and Figure 2 show the results of sex bias categorized by disease. The F-Prev was notably higher for endocrine, metabolic, blood and immune disorders (0.6647) as well as for HIV/AIDS and sexually transmitted infections (0.6319). Conversely, it was lower for substance abuse disorders (0.2907) and for total burden related to hepatitis B (0.4219).

**TABLE 4:** Sex bias by category disease.

Disease’s category	Global female prevalence fraction (F-Prev)	Measurement unit	Studies or participants, No.	Female participant fraction (F-Particip)	Sex bias
Cardiovascular diseases	0.5260	Studies	14	0.3704	−0.1556
		Participants	34,696	0.3751	−0.1509
Chronic respiratory diseases	0.5085	Studies	5	0.4222	−0.0863
		Participants	1,439	0.4997	−0.0089
Diabetes and kidney diseases	0.5207	Studies	7	0.4130	−0.1078
		Participants	21,059	0.4423	−0.0784
Digestive diseases	0.4856	Studies	3	0.4199	−0.0770
		Participants	493	0.4086	−0.0658
Endocrine, metabolic, blood and immune disorders	0.6647	Studies	12	0.3329	−0.2189
		Participants	15,649	0.4458	−0.3318
HIVAIDS and sexually transmitted infections	0.6319	Studies	3	0.1880	−0.5659
		Participants	1,048	0.0660	−0.4439
Maternal and neonatal disorders	0.5756	Studies	1	0.4658	−0.1098
		Participants	146	0.4658	−0.1098
Mental disorders	0.5236	Studies	5	0.4111	−0.1972
		Participants	1,012	0.3264	−0.1125
Musculoskeletal disorders	0.5733	Studies	5	0.5662	−0.0508
		Participants	2,847	0.6241	−0.0071
Neoplasm	0.5701	Studies	20	0.4504	−0.0410
		Participants	9,154	0.5290	−0.1197
Neurological disorders	0.5456	Studies	10	0.6775	0.0482
		Participants	6,486	0.5938	0.1319
Other infectious diseases	0.5039	Studies	4	0.3457	−0.1019
		Participants	1,128	0.4020	−0.1581
Respiratory infections and tuberculosis	0.4832	Studies	3	0.5219	0.0388
		Participants	3,196	0.5220	0.0387
Sense organ diseases	0.5079	Studies	5	0.5956	0.0406
		Participants	1,214	0.5485	0.0877
Skin and subcutaneous diseases	0.5184	Studies	8	0.4953	−0.0938
		Participants	1,692	0.4246	−0.0231
Substance use disorders	0.2907	Studies	2	0.4000	0.1502
		Participants	175	0.4409	0.1093
Total burden related to hepatitis B	0.4219	Studies	1	0.1795	−0.2424
		Participants	195	0.1795	−0.2424
Total burden related to hepatitis C	0.5192	Studies	3	0.2357	−0.2169
		Participants	628	0.3022	−0.2835
Urinary diseases	0.4486	Studies	1	0.7417	0.2932
		Participants	848	0.7417	0.2932
Total	0.5046	Studies	112	0.4540	−0.0506
		Participants	1,06,399	0.4280	−0.0766

When considering “studies” as the unit of measurement, females were significantly underrepresented (sex bias ≤ 0.05) in 12 out of 19 categories (63.2%), of the EOm. This was particularly notable in 144 HIV/AIDS and sexually transmitted infections (sex bias = −0.5659). There was an overrepresentation of females in three categories (15.8%) (sex bias ≥ 0.05), especially in the category of urinary diseases (sex bias = 0.2932).

When “participants” were considered as the unit of measurement, substantial female underrepresentation (sex bias ≤ 0.05) in the EOm was observed in 11 out of 19 categories (57.9%), such as HIV/AIDS and sexually transmitted infections (sex bias = −0.4439). In four categories (21.1%), there was female overrepresentation (Figure 2), for example in urinary diseases (sex bias = 0.2932).

Using both “studies” and “participants” as the unit of measurement, the degree of sex bias was increased in six categories. Similarly, the degree of sex bias was reduced in two categories.

### 3.3 Specific diseases

**FIGURE 3:**
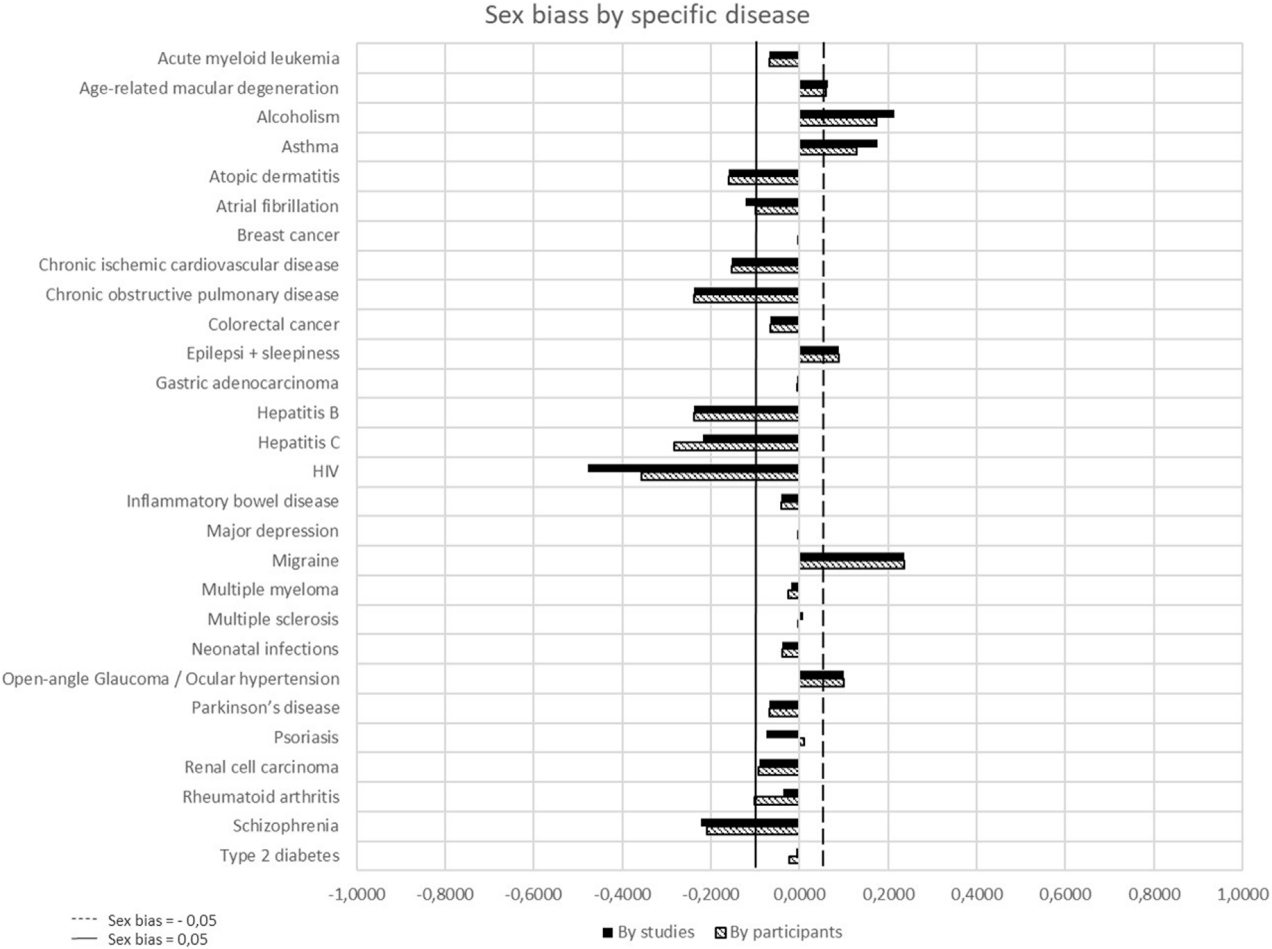
Sex bias by specific disease.

Out of the 112 EOm examined, only 58 studies (51.8%) focused on evaluating a specific disease listed in the database. Table 5 and Figure 3 show the results of sex bias by specific disease. Among these 58 EOm, there were a total of 52,381 participants, with 23,526 of them being female (44.9%). The F-Prev was notably higher for breast cancer (0.9897), rheumatoid arthritis (0.7100) and multiple sclerosis (0.6728). Conversely, it was lower for alcoholism (0.2269), gastric adenocarcinoma (0.2986) and renal cell carcinoma (0.3641).

**TABLE 5:** Sex bias by specific diseases.

Disease/disorder	Global female prevalence fraction (F-Prev)	Measurement unit	Studies or participants, No.	Female participant fraction (F-Particip)	Sex bias
Acute myeloid leukemia	0.4962	Studies	1	0.4275	−0.0687
		Participants	138	0.4275	−0.0687
Age-related macular degeneration	0.5783	Studies	2	0.6434	0.0651
		Participants	496	0.6371	0.0588
Alcoholism	0.2269	Studies	2	0.4409	0.2140
		Participants	175	0.4000	0.1731
Asthma	0.5169	Studies	2	0.6933	0.1764
		Participants	911	0.6454	0.1285
Atopic dermatitis	0.5910	Studies	1	0.4318	−0.1592
		Participants	308	0.4318	−0.1592
Atrial fibrillation	0.4927	Studies	2	0.3712	−0.1215
		Participants	4,528	0.3944	−0.0983
Breast cancer	0.9897	Studies	3	0.9916	0.0019
		Participants	936	0.9893	−0.0004
Chronic ischemic cardiovascular disease	0.4237	Studies	1	0.2699	−0.1538
		Participants	9,174	0.2699	−0.1538
Chronic obstructive pulmonary disease	0.5057	Studies	1	0.2688	−0.2369
		Participants	253	0.2688	−0.2369
Colorectal cancer	0.4220	Studies	1	0.3559	−0.0661
		Participants	236	0.3559	−0.0661
Epilepsy + sleepiness	0.4811	Studies	1	0.5694	0.0883
		Participants	72	0.5694	0.0883
Gastric adenocarcinoma	0.2986	Studies	1	0.2923	−0.0063
		Participants	2,135	0.2923	−0.0063
Hepatitis B	0.4180	Studies	1	0.1795	−0.2385
		Participants	195	0.1795	−0.2385
Hepatitis C	0.5192	Studies	3	0.3022	−0.2170
		Participants	628	0.2357	−0.2835
HIV	0.5441	Studies	3	0.0660	−0.4781
		Participants	1,048	0.1880	−0.3561
Inflammatory bowel disease	0.5076	Studies	1	0.4667	−0.0409
		Participants	15	0.4667	−0.0409
Major depression	0.6232	Studies	1	0.6229	−0.0003
		Participants	411	0.6229	−0.0003
Migraine	0.6328	Studies	1	0.8697	0.2369
		Participants	2,418	0.8697	0.2369
Multiple myeloma	0.4503	Studies	2	0.4318	−0.0184
		Participants	3,434	0.4257	−0.0245
Multiple sclerosis	0.6728	Studies	5	0.6799	0.0071
		Participants	2,496	0.6727	−0.0001
Neonatal infections	0.5040	Studies	1	0.4658	−0.0383
		Participants	146	0.4658	−0.0383
Open-angle Glaucoma/Ocular hypertension	0.4970	Studies	1	0.5962	0.0992
		Participants	577	0.5962	0.0992
Parkinson’s disease	0.4516	Studies	1	0.3846	−0.0670
		Participants	195	0.3846	−0.0670
Psoriasis	0.4989	Studies	7	0.4235	−0.0754
		Participants	1,384	0.5094	0.0105
Renal cell carcinoma	0.3641	Studies	2	0.2744	−0.0897
		Participants	790	0.2709	−0.0932
Rheumatoid arthritis	0.7100	Studies	4	06733	−0.0367
		Participants	2,175	0.6092	−0.1008
Schizophrenia	0.4748	Studies	4	0.2522	−0.2226
		Participants	601	0.2662	−0.2086
Type 2 diabetes	0.4826	Studies	3	0.4760	−0.0066
		Participants	16,506	0.4590	−0.0236
Total	0.5134	Studies	58	0.4615	−0.0519
		Participants	52,381	0.4600	−0.0534

When considering “studies” as the unit of measurement, there was a significant underrepresentation of females (sex bias ≤ 0.05) in EOm was observed in 13 out of the 28 diseases (46.4%), particularly notable in HIV (sex bias = −0.4781). On the contrary, eight specific diseases (28.6%) showed female overrepresentation, such as migraine (sex bias = 0.2369) or alcoholism (sex bias = 0.2140).

When “participants” was considered as the unit of measurement, substantial female underrepresentation (sex bias ≤ 0.05) in the examined diseases was noted in 13 diseases (46.4%), with HIV showing the most pronounced bias (sex bias = −0.4781). Conversely, six diseases displayed female overrepresentation, notably in alcoholism (sex bias = 0.1731) or asthma (sex bias = 0.1285).

In 10 diseases, the degree of sex bias was increased when “participants” was considered the unit of measurement.

## 4 Discussion

Several recent articles have addressed sex bias in clinical trials, but few have addressed this issue in relation to observational studies (National Library of Medicine, 2024). In the scientific literature, numerous articles highlight the necessity of stratify the results of clinical trials, observational studies and pharmacovigilance between males and females. Watson et al. analyzed the World Health Organization’s pharmacovigilance database (VigiBase) in 2019, which monitors adverse effects of medications in 131 countries. Their study revealed that women exhibited a higher proportion of severe adverse effects, –including death– compared to men (Watson et al., 2019). Similarly, several Phase III clinical trials have demonstrated distinct pharmacological actions between males and females across various therapeutic groups, such as antidiabetics, antineoplastic, antidepressants, antiretroviral, and certain vaccines like anthrax (McGill et al., 2013; Tejpar et al., 2018; Khan et al., 2007; Kumar et al., 2006; Pondo et al., 2014).

Moreover, a study conducted by Annaliese K. Beery and Irving Zucker showed that, even in animal research, females are often underrepresented. Their study revealed a male bias in eight of the ten fields surveyed, including neuroscience, physiology, pharmacology, among others (Beery and Zucker, 2011). In this context, our study is one of the first of its kind in pharmacological research, as there are no other articles evaluating sex bias in prospective follow-up observational studies with drugs (phase IV). While there is a substantial amount of literature discussing sex bias, it primarily focuses on preclinical stages or clinical trials, when the drug has not yet been approved. Given the importance to the evidence-based medicine, real-world evidence studies are opening avenues to utilize real-world data effectively and improving clinical decision-making, giving importance to sex equality in these studies (Taur, 2022).

Our study’s findings regarding sex bias reveal that despite existing regulations aimed at equalizing the representation of women in clinical trials, further efforts are needed to address sex bias in real-world evidence studies, such as the observational studies with drugs (Clayton and Tannenbaum, 2016; Sundari Ravindran et al., 2020; Woitowich and Woodruff, 2018). Notably, fields such as HIV, hepatitis B and C, and endocrine and metabolic diseases show lower representation of women, consistent with sex bias observed in clinical trials (Feldman et al., 2019). Previous studies of sex bias used either “studies” or “participants,” but not both, as measurement unit (Feldman et al., 2019). With “studies” as measurement unit, each study has an equal contribution to the overall sex bias estimate, regardless of study size. Our study demonstrates that sex bias is less pronounced when using “participants” as the measurement unit. This shows that small observational are more likely to underrepresent women than larger studies.

The discrepancy in sex bias between articles with “studies” versus “participants” as the measurement unit for mental disorders (−0.1125 vs. −0.1972) or endocrine, metabolic, blood and immune disorders (−0.3318 vs. −0.2189) is enough evidence that sex bias determined with both measurements units should be reported. It is worth noting that in observational studies it should be easier to recruit the sex that suffers most with the disease, result that is not found in our study.

While there is no unanimous agreement on the precise percentage indicating underrepresentation or overrepresentation in clinical research, it varies depending on study-specific criteria. Nonetheless, to remain consistent with other studies addressing sex bias similarly to ours, we have defined underrepresentation of women as a sex bias value equal to or less than 0.05 (Feldman et al., 2019).

Limitations of the present study include the analysis of sex bias without accounting for other potentially influential variables. Additionally, the lack of categorization of diseases by male and female prevalence made it challenging to access relevant data, particularly regarding the sex distribution of specific diseases. As noted in the article, the number of studies included in the “disease category” table exceeds that in the “specific diseases” table. This discrepancy arises from the difficulty of finding reliable literature on disease prevalence by sex, as not all diseases are included in the database used (GHDx). Furthermore, variations in disease prevalence between regions and age groups, such as colorectal and lung cancer, may have introduced further complexities into our analysis (Murthy et al., 2004). Grouping different diseases and disorders into distinct categories posed a potential source of bias, as the observational studies in our database may not align perfectly with the categorization in the Global Data Exchange (GHDx). Furthermore, another significant challenge was the absence of data, such as sex categorization in some of the EOm, introducing biases and limitations into our analyses, as we were unable to obtain a complete picture of sex representation in all the observational studies conducted in the southern region of Europe.

Despite our efforts to mitigate these biases and limitations, it is crucial to take these factors into account when interpreting our study’s findings and assessing implications for future research in this field. Additionally, further studies of this nature are needed to validate the theory’s applicability across different populations and settings.

### 4.1 Conclusion

Our study has demonstrated a significant sex bias within observational studies, mirroring patterns observed in clinical trials. Importantly, our findings highlight a pervasive underrepresentation of women across various disease categories (11 out of 19) and specific conditions (13 out of 28). Differences between sex bias estimates with “studies” vs. “participants” as measurement unit suggest that sex bias with both measures should be reported. The resemblance of sex bias between observational studies and clinical trials underscores the systemic nature of this issue within medical research. Despite efforts to promote both sexes inclusivity, our results emphasize the persistent challenges in achieving balanced sex representation in study populations.

Furthermore, the absence of categorization of diseases based on male and female prevalence poses a significant challenge in accessing pertinent data, particularly concerning the sex distribution of specific diseases. This highlights the need for enhanced efforts in establishing comprehensive databases integrating a sex perspective in disease prevalence. By systematically documenting and analyzing disease prevalence according to sex, such databases would not only provide valuable insights into the unique health experiences of women but also enable tailored interventions and policies to address sex-specific health disparities.

Addressing sex bias is essential for ensuring the validity and generalization of research findings, especially from real-world evidence, and to advance towards more equitable healthcare practices. It would be interesting to propose to the Regulatory Agencies the inclusion of sex as a variable of recruitment, to ensure the sex equity in this field. By acknowledging and actively combating sex bias, we can foster a more inclusive and evidence-based approach to medical research, ultimately improving healthcare outcomes for all individuals.

## Data Availability

The raw data supporting the conclusions of this article will be made available by the authors, without undue reservation.
